# Antibiotic Prescribing Among Inpatients with Ophthalmic Diagnoses: A Decade of Evidence from Two Private-Sector Indian Hospitals

**DOI:** 10.3390/antibiotics15080746

**Published:** 2026-07-31

**Authors:** Megha Sharma, Katherine Rennie, Hager Saleh, Shubhra Mehta, Manoj Mehta, Cecilia Stålsby Lundborg

**Affiliations:** 1 Department of Global Public Health, Health Systems and Policy: Medicines Focusing on Antibiotics, Karolinska Institutet, Tomtebodavägen 18 A, 171 77 Stockholm, Sweden; katherine.rennie5@nhs.net (K.R.); hager.saleh@ki.se (H.S.); cecilia.stalsby.lundborg@ki.se (C.S.L.); 2 Department of Pharmacology, Ruxmaniben Deepchand Gardi Medical College, Surasa, Ujjain 456006, Madhya Pradesh, India; 3 Department of Ophthalmology, Ruxmaniben Deepchand Gardi Medical College, Surasa, Ujjain 456006, Madhya Pradesh, India; shubhramehta@yahoo.com (S.M.); manojvkm@yahoo.com (M.M.)

**Keywords:** antibiotics, ophthalmology, infections, bacterial infections, AWaRe classification, prescribing patterns, trends, antibiotic resistance, India, low- and middle-income countries

## Abstract

Objectives: To describe and compare decadal antibiotic prescribing patterns and trends by diagnosis groups among ophthalmology inpatients at two private-sector hospitals in Central India. Methods: This observational surveillance study was conducted prospectively for a decade in the ophthalmology inpatient departments of a teaching hospital (TH) and a non-teaching hospital (NTH). Patient-level data on demographics, diagnoses, and antibiotic prescriptions were collected by nurses using a standardised form. Antibiotic utilisation was analysed using WHO’s Anatomical Therapeutic Chemical (ATC) codes and Defined Daily Doses (DDDs), as well as AWaRe groups (*Access*, *Watch*, *Reserve*). Diagnoses were classified into infectious and non-infectious categories, and surgical and non-surgical groups. Prescribing trends were analysed using descriptive statistics and linear regression models. Results: Of 7561 patients (TH: 7305; NTH: 256), antibiotics were prescribed to almost all inpatients (TH: 99%; NTH: 90%). Fluoroquinolones were the most prescribed (TH: 94%; NTH: 71%), predominantly by the oral route (73%). *Access*-group antibiotics were considerably under-prescribed relative to WHO targets (TH: 3%; NTH: 4%). *Watch*-group antibiotics comprised 97% (TH) and 96% (NTH) of all prescribed antibiotics, and their prescribing increased significantly over 10 years (*p* < 0.05). Conclusions: *Watch*-group fluoroquinolones dominated antibiotic prescribing at both hospitals and increased over the study period. *Access*-group antibiotics were substantially underutilised. These findings highlight an urgent need for context-specific antibiotic prescribing guidelines and stewardship programmes in ophthalmology settings in LMICs.

## 1. Introduction

The use of antibiotics is strongly associated with the development of antibiotic resistance (ABR), which is a serious and growing global threat [1,2]. ABR results in increased morbidity and mortality globally, longer hospital stays, and higher treatment costs. A 2015 WHO report showed that antibiotic use has rapidly increased globally, with consumption being particularly high in low- and middle-income countries (LMICs) [3]. Surveillance data on antibiotic prescribing is a vital first step for identifying areas where interventions are needed, informing prescribing guidelines, and rationalising antibiotic use across healthcare systems [3]. However, data from private-sector hospitals in LMICs remain limited, despite this sector serving a substantial share of the population.

Hospitals in LMICs are one of the major users of antibiotics [4]. The higher prevalence of infectious diseases and weaker regulatory infrastructure in LMICs create conditions that contribute to inappropriate antibiotic use and accelerate ABR [2,5]. Antibiotic stewardship programmes are therefore critically needed in these settings, but they must be supported by local prescribing data [6]. Without such baseline evidence, it is not possible to design, implement, or evaluate appropriate interventions.

The eye is a complex and delicate organ, and ABR may compromise the effectiveness of antibiotic therapy for ophthalmic infections, risking permanent visual impairment. Ophthalmic bacterial infections are common and are frequently treated empirically with antibiotics, often without microbiological confirmation. Whilst there is a growing body of literature on antibiotic prophylaxis in cataract surgery [7,8,9], there is a significant and specific gap in knowledge regarding patterns and long-term trends of antibiotic prescribing across the full spectrum of diagnoses encountered in ophthalmology inpatient departments, particularly in LMIC private-sector settings.

Antibiotics are used in ophthalmology settings for two main purposes: the treatment of bacterial infections and prophylaxis surrounding surgical procedures [7,8,9]. Consequently, both systemic and topical antibiotics constitute a routine component of ophthalmology inpatient care.

This study aims to compare and present the prescribing patterns and decadal trends in antibiotic prescribing by diagnosis types (bacterial infections or non-infectious conditions) among inpatients in the ophthalmology departments of two private-sector tertiary-care hospitals in Central India. Specifically, the objectives were to (i) analyse and compare the pharmacological and chemical subgroups of antibiotics prescribed in infectious and non-infectious categories among inpatients at the ophthalmology departments of both hospitals; (ii) evaluate adherence to the WHO AWaRe classification over time; and (iii) examine 10-year trends in antibiotic prescribing. Given that antimicrobial resistance develops as a consequence of cumulative antibiotic exposure over time, historical prescribing data remain highly relevant for interpreting current resistance challenges and for guiding future policy and guideline development. The long-term surveillance data from the ophthalmology specialty in this study will provide evidence to support the development of targeted antibiotic stewardship interventions and to guide rational antibiotic prescribing practices.

## 2. Results

### 2.1. Patient Demographics

In total, 7606 patients were admitted to the ophthalmology departments of both hospitals during the study period (TH: 7313; NTH: 293). As per the inclusion criteria, data from 7561 patients were included in the analysis (TH: 7305; NTH: 256; Figure S1).

The mean age of patients in the TH was 54.8 years, and in the NTH was 52.1 years; NTH patients were significantly younger (*p* < 0.05). The proportion of female patients was slightly higher at the NTH (57%) than at the TH (50%; *p* < 0.001). The median duration of hospital stay was significantly longer at the TH (4 days) than at the NTH (2 days; *p* < 0.001). Surgical procedures were performed on 57% of patients in the TH and 77% in the NTH (*p* < 0.001). The TH recorded 344 distinct diagnoses over the study period, compared with 54 at the NTH.

**Figure 1:**
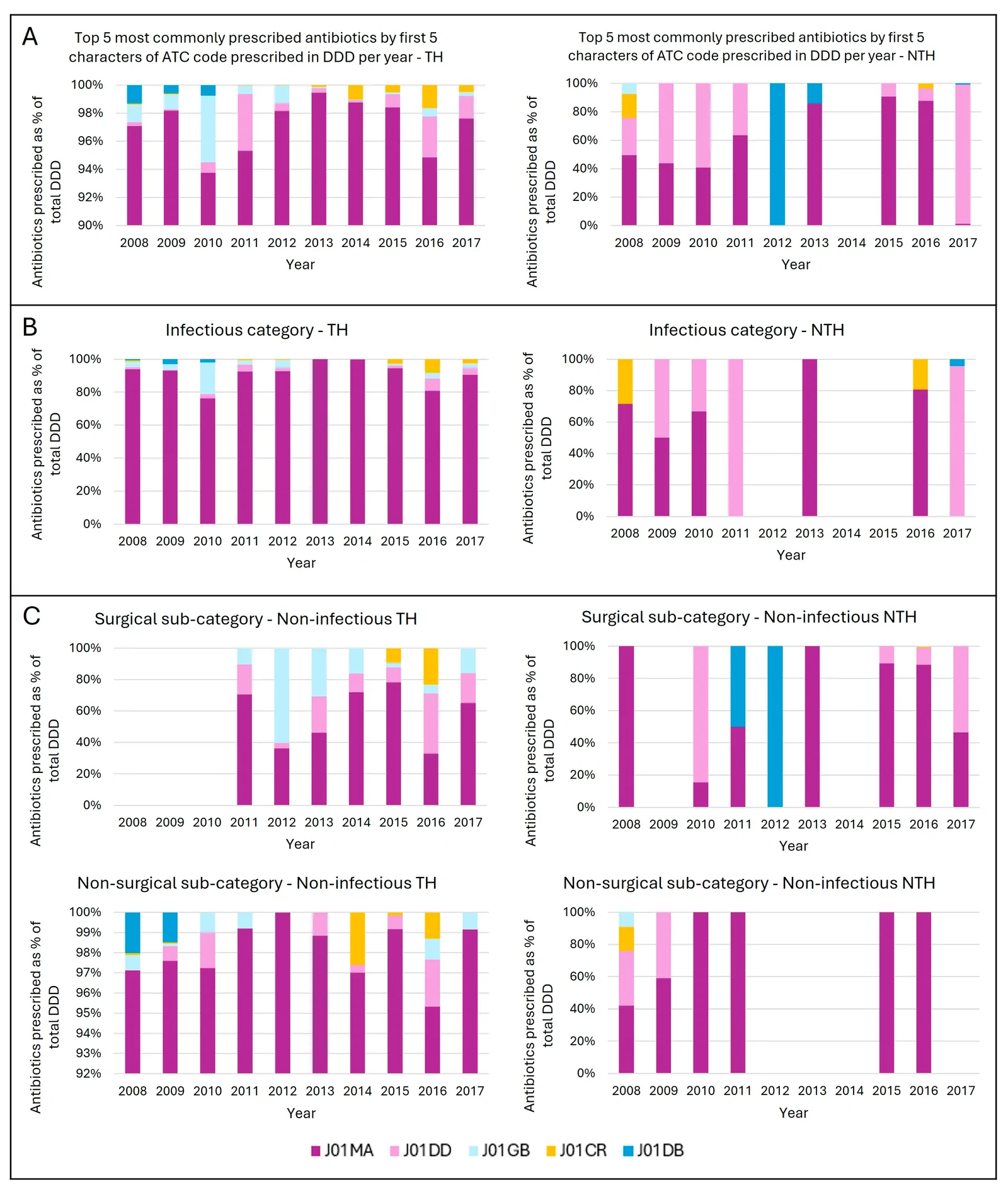
The percentages of DDDs of the five most prescribed antibiotics sorted by the chemical subgroup of the ATC code in ophthalmology departments of the TH and the NTH over a decade. (**A**) Top five most prescribed antibiotics by the first five characters of the ATC code prescribed in DDD per year. (**B**) Top five most prescribed antibiotics by the first five characters of the ATC code prescribed in DDD per year-infectious categories. (**C**) Top five most prescribed antibiotics by the first five characters of the ATC code prescribed in DDD per year- non-infectious categories. J01MA = fluoroquinolones, J01DD = third-generation cephalosporins, J01GB = aminoglycosides, J01CR = combinations of penicillin, and J01DB = first-generation cephalosporins. DDD—Defined Daily Dose, NTH—non-teaching hospital, TH—teaching hospital.

Microbiological culture and sensitivity tests were ordered in 21% of TH patients and 2% of NTH patients. Patient distribution by year is detailed in Figure 1; in the TH, the highest admissions occurred in 2016 (16%), 2014 (14%), 2015 (13%), and 2013 (12%). In the NTH, over half of the patients were admitted in 2016 (55%) and 2017 (17%).

### 2.2. Antibiotic Prescribing Overview

Antibiotics were prescribed to 99% of TH inpatients and 90% of NTH inpatients (*p* < 0.001). The median post-discharge antibiotic duration was longer in the TH than in the NTH (TH: 6 days, range: 1–44; NTH: 4 days, range: 1–46; *p* < 0.001). Most antibiotics prescribed in both hospitals were administered orally (TH: 70%; NTH: 76%; Table S1A,B) and as eye drops (TH: 30%; NTH: 24%), with less than 1% administered parenterally in either hospital. The most frequently prescribed pharmacological subgroup was quinolones (J01M) in both the infectious (TH: 86%; NTH: 51%) and non-infectious (TH: 96%; NTH: 79%) indication categories (*p* < 0.001; Figure 1B,C). At discharge, 85% of patients were prescribed antibiotics in the TH and 90% in the NTH (*p* < 0.001). Quinolones remained the most prescribed subgroup in both hospitals (TH: 95%; NTH: 76%, *p* < 0.001), followed by other beta-lactam antibacterials for the infectious (J01D; TH: 5%; NTH: 21%) and non-infectious categories (TH: 2%; NTH: 16%; *p* < 0.001).

Details of the distribution of all prescribed antibiotics are provided in the Supplementary Tables (Table S1A,B). Overall, the most prescribed antibiotic chemical subgroup was fluoroquinolones (J01MA; TH: 94%; NTH: 71%, *p* < 0.001, Figure 1A, and the most prescribed antibiotic substances were ciprofloxacin (TH: 87%; NTH: 53%) and moxifloxacin (TH: 9%; NTH: 22%). Other antibiotics were prescribed to a lesser extent (TH: 4%; NTH: 25%). Figure 1B,C show the chemical subgroups of the prescribed antibiotics for patients in the infectious and non-infectious categories, respectively. Comparing the two categories in the TH, a greater proportion of fluoroquinolones (J01MA) was prescribed to patients in the infectious category, whereas higher proportions of other beta-lactam antibiotic subgroups were prescribed to patients in the non-infectious category (*p* < 0.001). In the NTH, a substantial proportion of patients were prescribed fluoroquinolones (J01MA) and third-generation cephalosporins (J01DD) in both categories. Compared with patients in the infectious categories at both hospitals, the TH had a higher proportion of fluoroquinolones (J01MA; *p* < 0.001), and the NTH had a higher proportion of third-generation cephalosporins (J01DD; *p* < 0.001).

### 2.3. Antibiotic Prescribing in Infectious and Non-Infectious Categories

Patients were categorised into an infectious group (TH: 908; NTH: 26) and a non-infectious group (TH: 6343; NTH: 228). Antibiotics were prescribed to 99% of infectious-category patients in the TH and 90% in the NTH (*p* < 0.001), while prescribing in the non-infectious category was higher in both hospitals (TH: 99%; NTH: 96%, *p* < 0.001). The mean duration of antibiotic prescribing was longer in infectious-category patients compared with non-infectious patients (TH: 6.8 vs. 5.6 days, *p* < 0.001; NTH: 3.7 vs. 3.0 days, *p* = 0.06). In the non-infectious category, the majority of patients had surgical procedures (TH: 99%; NTH: 83%); 62% of patients with a non-infectious indication (3908/6343) in the TH and 83% of patients (190/228) in the NTH had a record of a surgical procedure, and the rest were not operated on (Figure S1).

### 2.4. Diagnosis-Based Prescribing Trends

**Figure 2:**
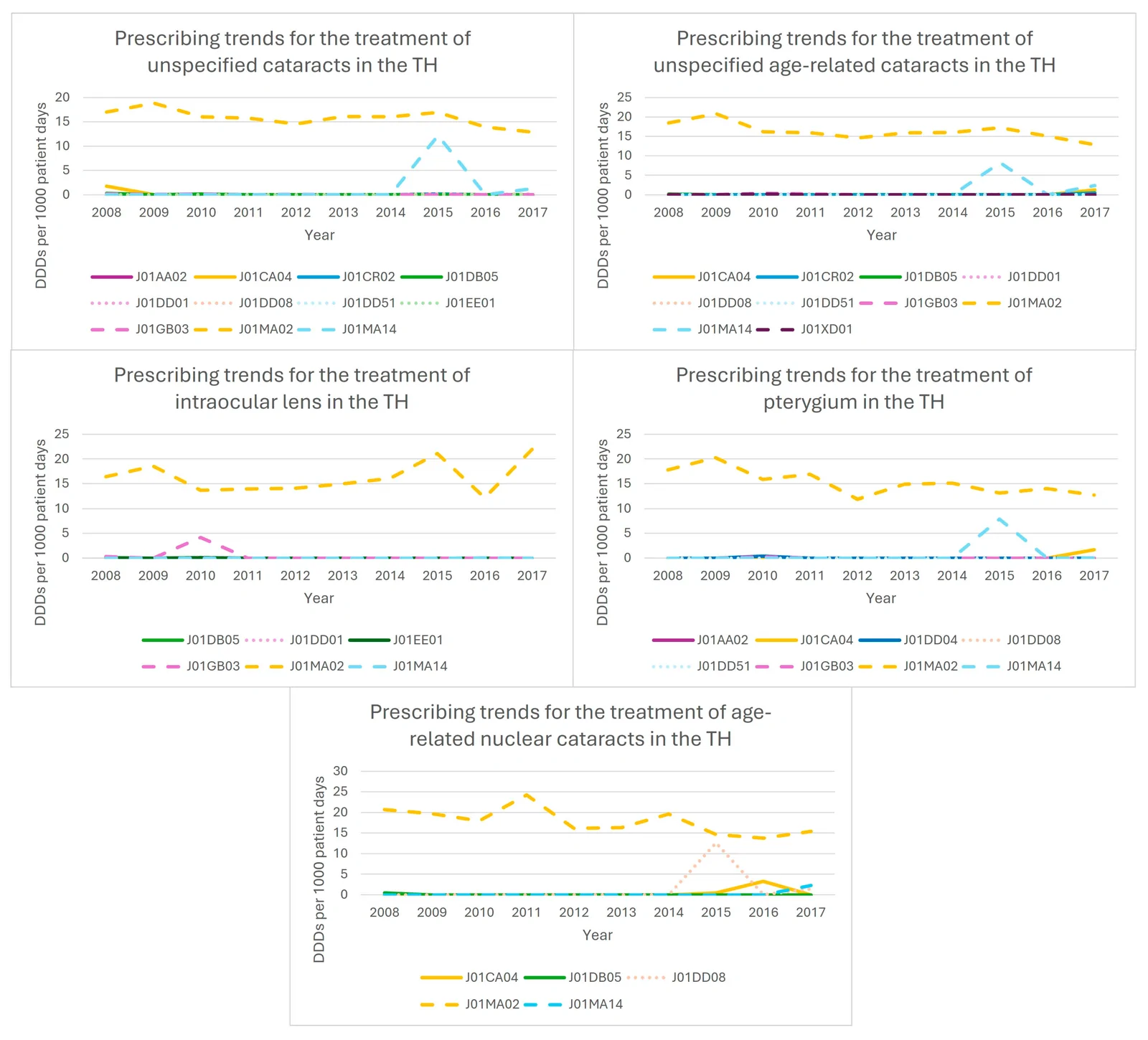
Antibiotic prescribing trends for the most common diagnoses in the ophthalmology departments of the TH over a decade. J01AA02 = Doxycycline, J01CA04 = Amoxicillin, J01CR02 = Amoxicillin + Clavulanic acid, J01DB05 = Cefadroxil, J01DD01 = Cefotaxime, J01DD04 = Ceftriaxone, J01DD08 = Cefixime, J01DD51 = Moxifloxacin + Methylcellulose, J01EE01 = Sulfamethoxazole + Trimethoprim, J01GB03 = Gentamicin, J01MA02 = Ciprofloxacin, J01MA14 = Moxifloxacin, and J01XD01 = Metronidazole. DDD—Defined Daily DoseTH—teaching hospital.

**Figure 3:**
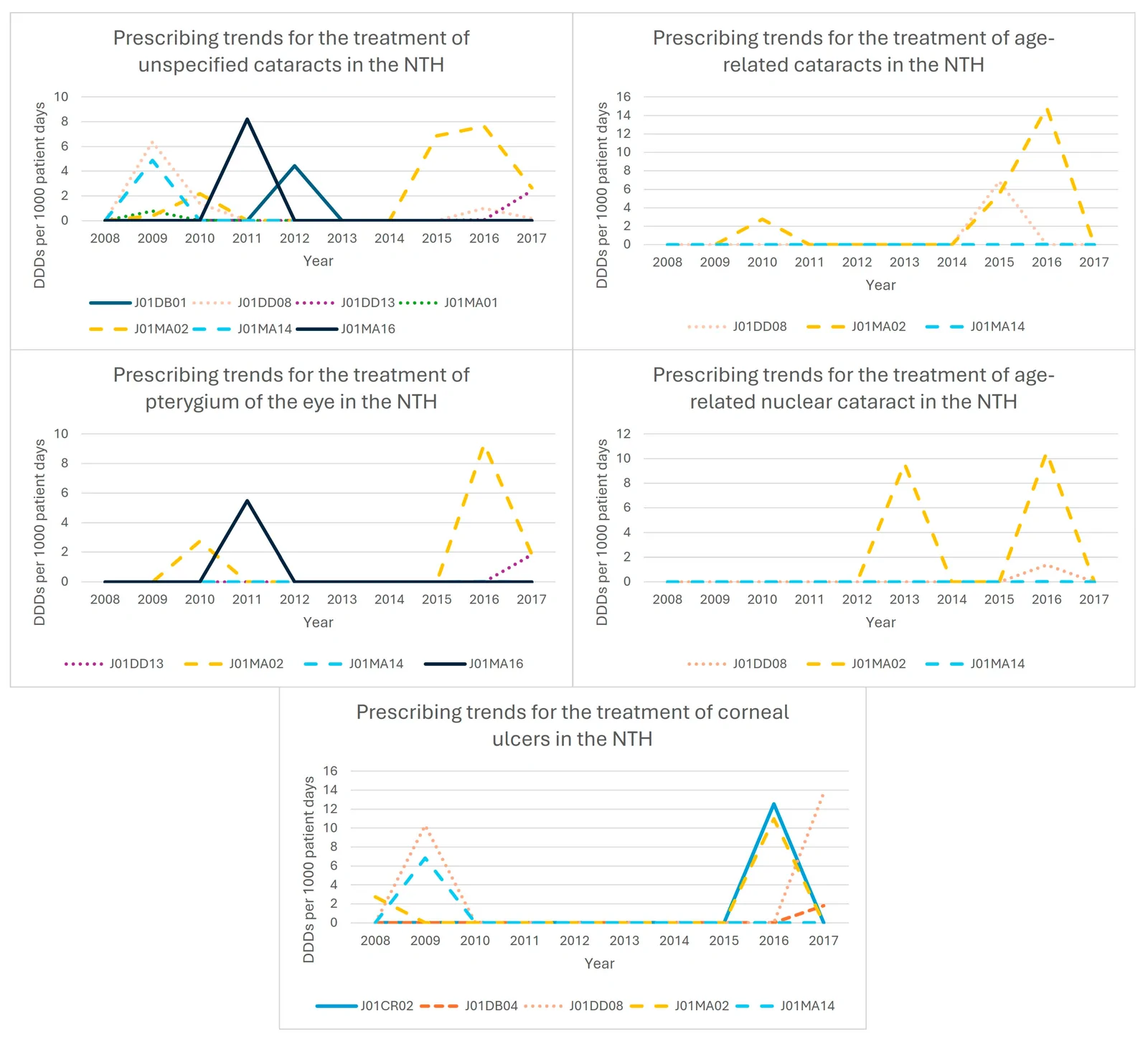
Antibiotic prescribing trends for the most common diagnoses in the ophthalmology departments of the NTH over a decade. J01CR02 = Amoxicillin + Clavulanic acid, J01DB01 = Cefalexin, J01DB04 = Cefazolin, J01DD08 = Cefixime, J01DD13 = Cefpodoxime, J01MA01 = Ofloxacin, J01MA02 = Ciprofloxacin, J01MA14 = Moxifloxacin, and J01MA16 = Gatifloxacin. DDD—Defined Daily Dose, NTH—non-teaching hospital.

The five most common diagnoses at the TH were unspecified cataract, unspecified age-related cataract, intraocular lens procedures, pterygium, and age-related nuclear cataract (Figure 2). Ciprofloxacin was the most prescribed antibiotic across all five diagnoses throughout the study period, with a notable increase in moxifloxacin (J01MA14) prescribing from 2015 onward for cataract and pterygium diagnoses. At the NTH, no single consistent trend was found across the five most common diagnoses (Figure 3).

### 2.5. Trends of the Most Prescribed Chemical Subgroups of Antibiotics

**Figure 4:**
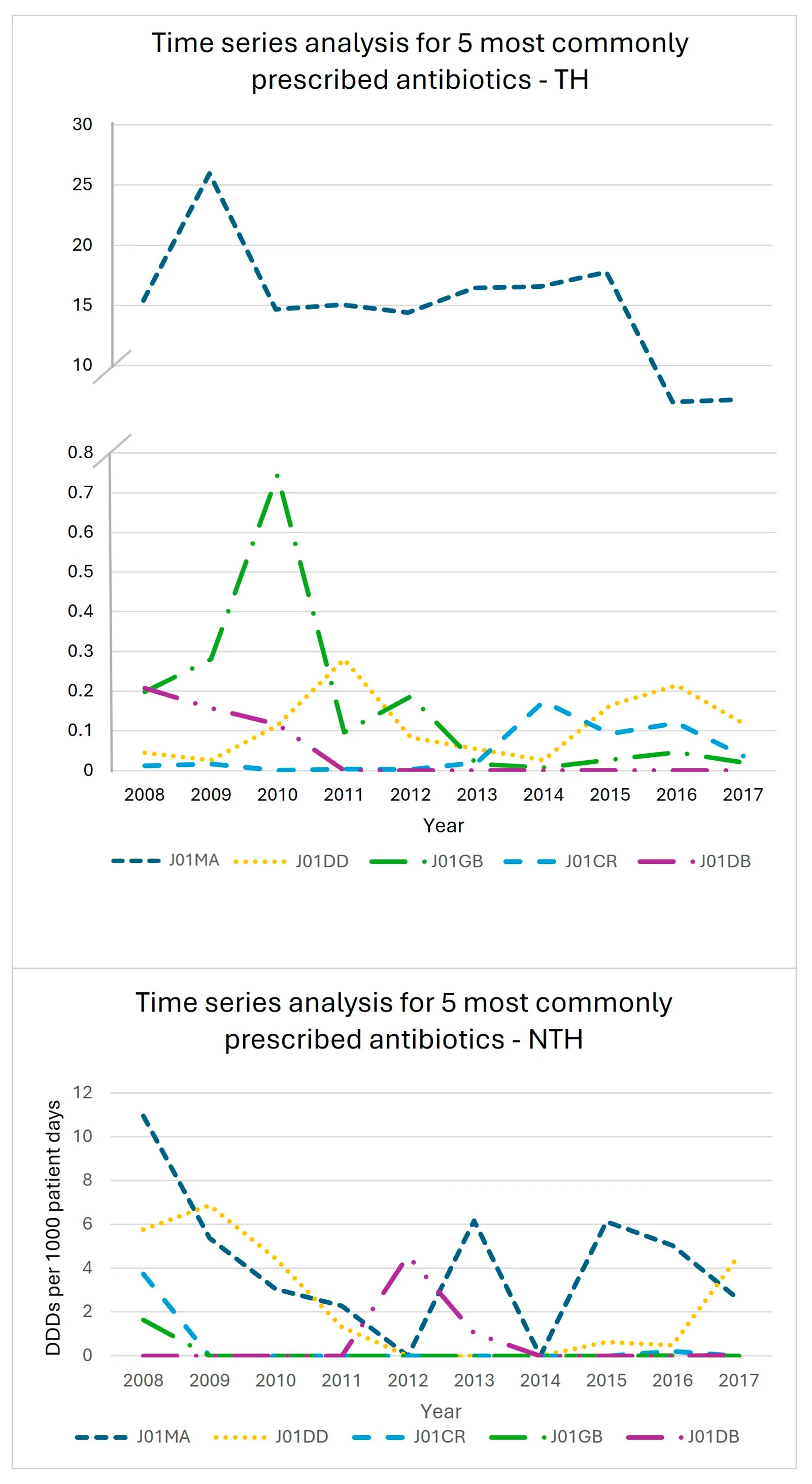
Line graphs displaying the five most prescribed antibiotics in the ophthalmology departments of the TH and the NTH, listed in descending order of the most prescribed antibiotic, over a decade. J01MA = fluoroquinolones, J01DD = third-generation cephalosporins, J01GB = aminoglycosides, J01CR = combinations of penicillin, and J01DB = first-generation cephalosporins. Due to the high proportion of J01MA prescribed in the TH (over 90% of all antibiotics each year), the graph uses a split y-axis to show time-series changes in the less frequently prescribed antibiotics. DDD—Defined Daily Dose, NTH—non-teaching hospital, TH—teaching hospital.

At the TH, fluoroquinolone (J01MA) DDDs per 1000 patient-days peaked in the first study year and declined from 2015 onwards. Other antibiotic subgroups remained consistently low (0–0.3 DDDs/1000 patient-days). At the NTH, prescribing across subgroups fluctuated without a consistent directional trend, suggesting variable prescribing behaviour over time (Figure 4).

### 2.6. Adherence to the AWaRe Antibiotic Guidelines

**Figure 5:**
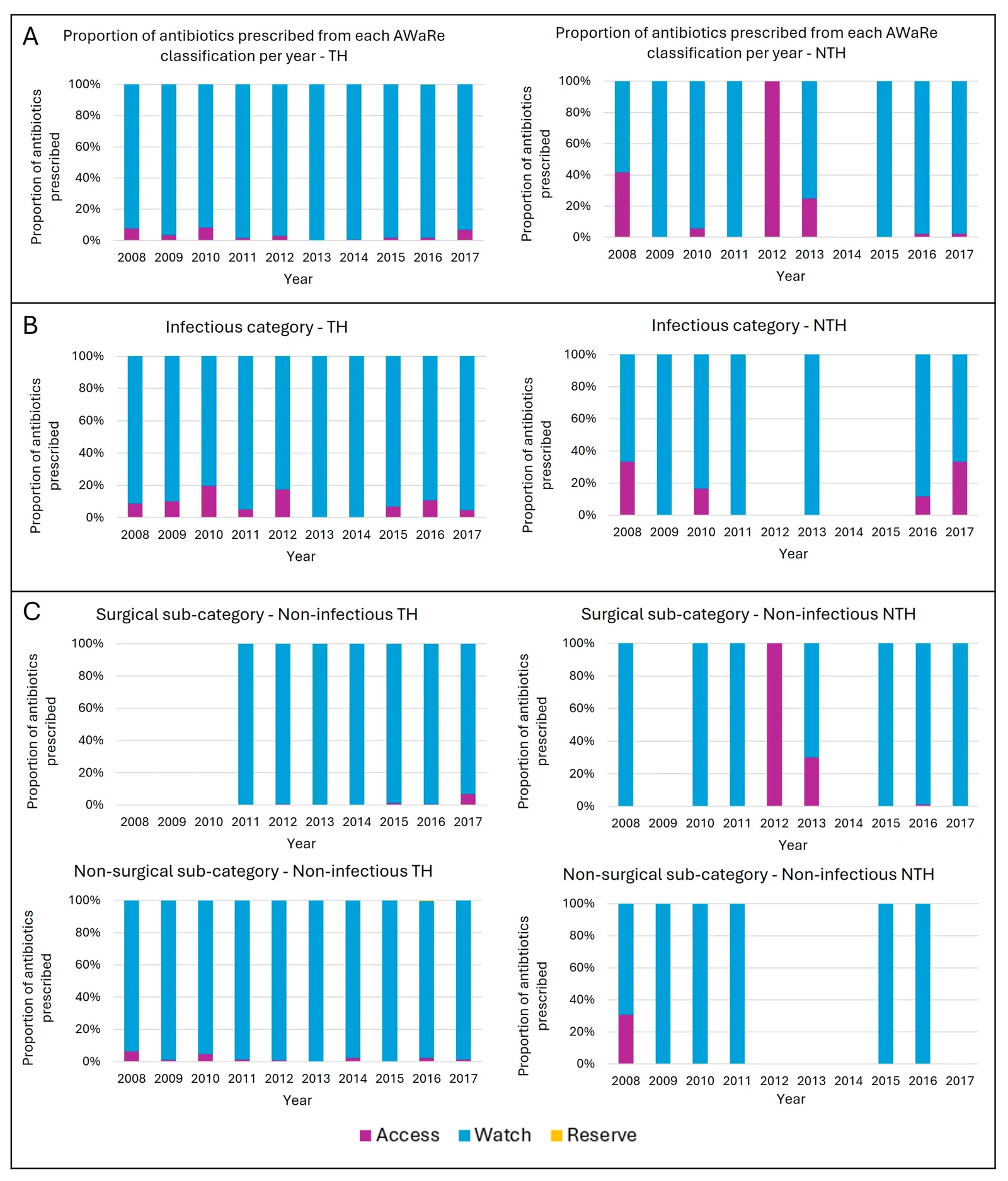
The distribution of prescribed antibiotics in AWaRe classification categories linked to overall and selected diagnosis categories in ophthalmology departments of the study hospitals over a decade. (**A**) Year-wise percentage of all prescribed antibiotics based on the AWaRe classification in the ophthalmology departments of the TH and the NTH. Only 1 *Reserve*-group antibiotic was prescribed during the 10-year period. (**B**) Year-wise percentage of the prescribed antibiotics based on the AWaRe classification for the infectious categories in the ophthalmology departments of TH and NTH. (**C**) Year-wise percentage of the prescribed antibiotics for the AWaRe classification in the surgical and non-surgical sub-categories of the non-infectious categories in the ophthalmology departments of the TH and the NTH. Antibiotics prescribed for TB were not included in this analysis because TB prescribing guidelines specify a set of antibiotics that are not listed in the AWaRe classification.

Figure 5 shows the distribution of prescribed antibiotics in AWaRe categories. *Watch*-group antibiotics dominated prescribing throughout the study period, comprising 97% of all antibiotic prescriptions at the TH and 96% at the NTH in Figure 5A. *Access*-group antibiotics accounted for only 3% of total prescriptions in the TH and 4% in the NTH. *Reserve*-group antibiotics were prescribed in only one instance at the TH and zero at the NTH in Figure 5A. The proportion of *Watch* antibiotics prescribed increased significantly over the study period at both hospitals (TH: 92% in 2010 to 100% in 2013–2014; NTH: 58% in the first study year to 100% in 2009 and 2015, *p* = 0.03; Figure 5A. Among patients with infectious indications, *Access* antibiotics were prescribed in 8 of 10 study years (except 2013 and 2014) at the TH and in 4 years (2008, 2010, 2016, and 2017) at the NTH (*p* < 0.001; Figure 5B. In both hospitals, *Watch* antibiotics predominated in both surgical and non-surgical sub-categories in Figure 5C.

## 3. Discussion

To the best of our knowledge, this is the first study to present and compare antibiotic prescribing patterns and long-term trends in the ophthalmology departments of Indian private-sector hospitals. Although prior publications from this larger research have examined prescribing across other departments and disease categories [10,11,12,13], none have addressed ophthalmology-specific prescribing trends, underscoring the novelty and significance of the present study. The generalisability of these findings is supported by the large sample size, the 10-year prospective study period, and the comparator design across teaching and non-teaching hospital settings.

### 3.1. Antibiotic Prescribing over the Decade

The present study highlights that antibiotics were prescribed to a majority of patients admitted to the ophthalmology wards (TH: 99%; NTH: 90%). In both hospitals, the most commonly prescribed antibiotic class for inpatients was fluoroquinolones (J01MA: TH: 95%; NTH: 76%). Additionally, the proportion of prescriptions for *Watch* antibiotics increased in both hospitals over the study period. These high prescribing rates across a predominantly surgical, non-infectious patient cohort indicate that antibiotic use is driven more by prophylactic convention than by documented infection, a pattern with significant implications for Watch antibiotic stewardship.

Eight of the top ten diagnoses in both hospitals were non-infectious aetiologies, and a majority of those required surgical procedures. In both hospitals, the most common diagnosis was unspecified cataract. This was consistent with the results of a 2011 study by Vashist et al., which found that the prevalence of non-operated cataract in people aged 60 or over was 58% in Northern India and 53% in Southern India [14].

A slight increase in the average number of prescribed antibiotics per patient was observed in both hospitals over 10 years (TH: 0.02 antibiotics per patient/year; NTH: 0.01 antibiotics per patient/year). It was not possible to find studies in ophthalmology departments comparing teaching and non-teaching hospitals, thereby establishing a baseline for future studies and policymakers. A 5-year study by Silfwerbrand et al. in otorhinolaryngology departments showed that a greater proportion of patients were prescribed antibiotics in these departments of the TH (89%) than in the NTH (82%) [15]. These figures are comparable; however, they are lower than the prescribing figures found in the present study (TH: 99%; NTH: 90%).

Antibiotics were prescribed most frequently to inpatients in both the surgical and non-surgical, non-infectious diagnosis groups. One reason for prescribing antibiotics in the non-surgical groups might be the minimal utilisation of microbiological (bacterial) culture and susceptibility testing facilities. However, high proportions of antibiotic prescribing in the surgical procedure groups may be due to guidelines recommending prophylactic antibiotics for these patients to reduce the risk of healthcare-associated infections [15,16].

### 3.2. High Proportion of Oral Antibiotic Use

A key finding of this study is the predominance of systemic oral antibiotic formulations (TH: 70%; NTH: 76%) relative to topical eye drops (TH: 30%; NTH: 24%). This is clinically important because topical formulations achieve higher local drug concentrations at the site of ocular infection, whereas oral or parenteral antibiotics expose the systemic circulation to unnecessary antibiotic pressure, thereby contributing disproportionately to the development of ABR. The dominance of systemic prescribing may reflect several factors: prescribing habits developed in the absence of formal ophthalmology-specific antibiotic guidelines; the assumption that systemic antibiotics provide additional prophylactic benefit in the perioperative setting; the perceived ease of prescribing familiar systemic agents; or the limited availability and higher cost of specialist ophthalmic antibiotic drops. Additionally, in settings where formal antibiotic stewardship guidance is lacking, prescribers may default to broad-spectrum oral agents as a precaution, particularly for postoperative patients and those with comorbidities. These findings underscore the urgent need for evidence-based, locally contextualised guidelines that specify when oral versus topical therapy is appropriate in ophthalmology.

### 3.3. Fluoroquinolone Prescribing and Watch Antibiotics’ Dominance

While fluoroquinolones dominated in both hospitals, the NTH showed greater year-to-year variation in the leading chemical subgroup, a pattern that may reflect susceptibility to external prescribing influences. This variation in the NTH could be due to pharmaceutical company representatives’ influence. One hypothesis is that pharmaceutical company representatives may differentially influence prescribing at the NTH; evidence from comparable settings suggests that pharmaceutical marketing can shape prescriber behaviour [17]. However, as this study collected no data on physician–industry interactions or prescriber behaviour, this explanation remains hypothetical and should be explicitly understood as a hypothesis warranting investigation in future research.

Third-generation cephalosporins (J01DD) were also among the most prescribed antibiotics, particularly in the NTH. These results are comparable to those of a few studies from intensive care units in India, orthopaedic departments, and community hospitals, in which high proportions of fluoroquinolones and third-generation cephalosporins were prescribed [13,18,19,20]. These chemical subgroups are commonly prescribed due to their broad-spectrum properties [21].

Within ophthalmology specifically, fluoroquinolone predominance has also been noted in an Indian tertiary ophthalmology department [22], partly reflecting the global practice of fluoroquinolone-based prophylaxis against postoperative endophthalmitis in cataract and intraocular surgery [7,8,9]. Across broader Indian healthcare settings, *Watch* antibiotics, the category to which fluoroquinolones belong, account for an estimated 36–48% of antibiotic consumption [23], in contrast to the proportions observed here.

Notably, fluoroquinolones are classified in the *Watch* group under the WHO AWaRe classification, indicating a higher potential for resistance and the need to reserve them for specific clinical indications [24]. The proportion of *Watch*-group antibiotics prescribed (TH: 97%; NTH: 96%) far exceeds the WHO target of ≤40% *Watch* antibiotic use, and this proportion increased over the study period, a trend that is particularly noteworthy from a resistance stewardship perspective.

### 3.4. Under-Prescribing of Access Antibiotics and Minimal Culture Practice

*Access*-group antibiotics accounted for only 3–4% of prescriptions in both hospitals, compared with the WHO target of ≥60% [24]. This represents a serious gap. The underutilisation of microbiological culture and sensitivity testing (culture ordered in only 21% of TH patients and 2% of NTH patients) is a likely contributing factor. Without culture guidance, clinicians tend to default to broad-spectrum agents, increasing *Watch* antibiotic use and limiting the opportunities to target *Access* antibiotic therapy. Strengthening laboratory infrastructure and integrating culture testing into routine clinical pathways for suspected ocular infections would be a critical step towards more rational prescribing. On the other hand, *Reserve* antibiotics accounted for less than 0.01% of the total antibiotics prescribed in both hospitals. It is encouraging that prescribers at the study hospitals are saving the *Reserve* antibiotics for future use.

A notable exception to the overall pattern of *Watch* antibiotic dominance is the transient increase in *Access* antibiotic use visible in Figure 5 around 2012, followed by a return to near-exclusive *Watch* prescribing in subsequent years. Although the study data do not permit definitive attribution, several explanations exist. This shift may reflect a temporary change in prescribing personnel during that period, with individual prescribers differing in their antibiotic preferences. Alternatively, it may reflect a higher proportion of patients with bacterial infections for which *Access* antibiotics (such as cefalexin or amoxicillin-clavulanate) were the empirical choice, or a transient supply disruption affecting fluoroquinolone availability.

All antibiotics prescribed at both study hospitals were listed in the AWaRe classification and the WHO Essential Medicines List. This shows that these hospitals have access to essential medicines. A study by Kar et al. reports that in 2010, one-third of the world’s population lacked access to essential medicines [25]. Therefore, the fact that these hospitals in India have access to these essential medicines should be seen as an advantage. However, this contrasts with a study conducted in an Indian ophthalmology department by Ahluwalia et al., which found that only 37% of medications prescribed there were on the WHO Essential Medicines List [22].

### 3.5. Global Implications for ABR

Previously published studies from LMICs in this field are largely short-term, cross-sectional investigations with limited ability to evaluate trends over time [26,27]. Therefore, longitudinal data gaps, particularly in low- and middle-income countries, enhance the scientific value of the present study. As such, these results offer baseline evidence on prescribing practices, which are otherwise grossly missing, and can inform future studies, support comparisons over time, and contribute to the development and evaluation of antimicrobial stewardship interventions and policies.

The findings of this study, although specific to two private-sector hospitals within one district of India, are consistent with patterns reported in comparable LMIC settings and may be illustrative of prescribing dynamics in similar contexts. Fluoroquinolone resistance in ocular pathogens, including Staphylococcus aureus, Pseudomonas aeruginosa, and Enterobacteriaceae, is well documented globally and is directly linked to the volume of fluoroquinolone use [24]. The high and increasing *Watch* antibiotic prescribing trends observed in this study are consistent with the broader pattern of Watch antibiotic overuse documented in the ABR literature. Reporting these patterns contributes to the body of LMIC-specific surveillance evidence needed to inform stewardship efforts in comparable settings, consistent with the WHO Global Action Plan on ABR [3].

### 3.6. Recommendation for Future Intervention

The longitudinal data gaps, particularly in low- and middle-income countries, enhance the data’s scientific value. Considering these results as baseline evidence on prescribing practices, which are otherwise grossly missing, informs future studies, supports comparisons over time, and contributes to the development and evaluation of antimicrobial stewardship interventions and policies.

Based on these findings, we make the following recommendations: (i) the development and implementation of evidence-based, ophthalmology-specific antibiotic prescribing guidelines tailored to local resistance patterns and aligned with WHO AWaRe classifications; (ii) the establishment or strengthening of antibiotic stewardship programmes within these and similar private-sector hospitals, with active prescriber education; (iii) the expansion of microbiological culture and sensitivity testing infrastructure and the integration of culture results into treatment decision-making; (iv) prospective monitoring of AWaRe antibiotic adherence as a performance indicator at the hospital level; (v) multi-centre studies involving larger numbers of private-sector ophthalmology departments in LMICs to further characterise prescribing patterns and the impact of stewardship interventions; and (vi) implementation of an electronic medical record system (EMRS) to facilitate future studies as described in studies of our research group [28,29].

### 3.7. Strengths and Limitations

The key strengths of this study include its prospective design, consistency in data collection methodology across both hospitals, and the decadal data, which enable longitudinal trend analysis. The study provides a novel, department-specific, diagnosis-stratified analysis using baseline data to inform future policy and stewardship interventions. The prospective study design: The 10-year prospective data collection facilitated a large sample size over a long period, enabling generalisation. The data collection method, although time- and resource-intensive, can be used in other similar settings without EMRs.

Limitations include a major infrastructural inadequacy, the use of paper records, and the absence of computerised patient records (EMRs) in these resource-constrained settings. This made the whole process cumbersome and substantially delayed the analyses and dissemination of the results. It also hindered access to the medication history of previous treatments, for justifying the antibiotic prescriptions for non-infectious diagnoses. The absence of culture testing data during the first 3 years of the study, resulting in underreporting the proportion of patients who were ordered cultures; the smaller sample size at the NTH compared with the TH limits the statistical power of between-hospital comparisons; eye-drop prescriptions may be under-recorded in both hospitals, resulting in underreporting; and the study was conducted in two hospitals in a single district of India, which may limit generalisability to other geographic settings, although the private-sector hospital design and data collection approach are broadly representative of comparable LMIC settings. The time-series analysis is based on ten annual observations, which limits inferential statistical power; temporal findings are therefore presented as descriptive trends and should not be interpreted as definitive evidence of causal change.

## 4. Materials and Methods

### 4.1. Study Setting

This prospective observational study was conducted in the ophthalmology inpatient departments of two tertiary-care, private hospitals in the Ujjain district of Madhya Pradesh, Central India, over 10 consecutive years (2008 to 2017). One hospital is located in a rural setting and is affiliated with a medical college; hence, it is referred to as the teaching hospital (TH). The other hospital is in an urban area and is referred to as the non-teaching hospital (NTH) [10,11]. The TH has 760 beds, and the NTH has 400 beds [30]. Neither hospital had computerised patient medical record systems; therefore, data were collected manually by nursing staff.

Both study hospitals are managed by a not-for-profit trust that serves both rural and urban populations. At the TH, medical services and medications are provided free of charge to both inpatients and outpatients. At the NTH, service costs are subsidised but paid out-of-pocket by patients [10]. Medical representatives are prohibited from visiting the prescribers at the TH but are permitted at the NTH [11]. The local list of essential medicines at the TH was available but was not fully implemented during the study period. There were no local antibiotic prescribing guidelines available at either hospital during the study period.

### 4.2. Study Design and Data Collection

This observational study was part of a larger project documenting antibiotic prescribing patterns and trends in other departments using the same methodology [10,11,12,13]. However, none of these prior publications analysed prescribing patterns or diagnosis-based prescribing trends across a 10-year period from the ophthalmology departments [10,11,12,13]. Due to the absence of computerised medical records, data were collected prospectively by trained nursing staff who underwent repeated training to use a validated, locally designed data collection tool. The data mainly included patients’ demographic details, diagnoses, and antibiotics prescribed during the hospital stay and at discharge. Each patient was assigned a unique identification number to maintain full confidentiality during data entry in EpiData version 3.1.

### 4.3. Inclusion and Exclusion Criteria

A census sampling strategy was adopted; data were collected from all patients admitted to the ophthalmology department during the study period. The inclusion criteria for analyses comprised adult patients (aged >17 years) admitted to the ophthalmology ward for at least one overnight stay, with complete and adequately recorded data in the data collection tool. Both postoperative patients (e.g., those undergoing cataract surgery, pterygium excision, or other ophthalmic surgical procedures) and non-surgical patients were included. Patients with incomplete data (i.e., missing diagnoses or antibiotic prescription details) were excluded from the analysis (Figure S1). Since the study adopted an all-inclusive census design covering all patients admitted during the 10-year period, no formal sample size calculation was required or performed. The sampling frame encompassed the entire eligible patient population, ensuring the dataset was representative of the full range of clinical presentations managed at these ophthalmology departments.

### 4.4. Patient and Public Involvement

Patients were not involved in the design, conduct, reporting, or dissemination plans of our research.

### 4.5. Data Analysis

The datasets were exported into Stata version 16.1 (Stata Corp., College Station, TX, USA) and Microsoft Excel Online version 2101 to generate the final datasets for analysis. Anatomical Therapeutic Chemical (ATC) codes and Defined Daily Doses (DDDs) were added to the data based on the WHO ATC/DDD methodology of the WHO Collaborating Centre for Drug Statistics Methodology [13,31]. International Classification of Diseases (ICD-10) codes were used to code the diagnoses [32]. In-depth analyses were conducted at the pharmacological subgroup (ATC third level), chemical subgroup (ATC fourth level), and chemical substance (ATC fifth level). For antibiotic eye drops, the dose in grams was calculated according to the WHO recommendation that 0.1 mL is 1 dose [33].

DDDs were calculated for all antibiotic formulations (syrup, tablet, eye drop, etc.) using the formula:

$$DDD\;per\;prescription=\frac{dose\;in\;grams\;\times \;frequency\;}{WHO\;DDD\;for\;the\;prescribed\;antibiotic}$$

All prescribed antibiotics were further classified according to the WHO AWaRe (*Access*, *Watch*, *Reserve*) classification of antibiotics [29]. Diagnoses were classified into two categories: (1) infectious diagnoses of bacterial origin, and (2) non-infectious diagnoses. The non-infectious category was further subcategorised into surgical and non-surgical subgroups based on whether a surgical procedure was performed during the hospital stay. This categorisation was guided by available international antibiotic prescribing guidelines and recommendations for prophylactic antibiotic use in surgical patients [28].

Descriptive statistics were used to summarise demographic and prescribing variables. Categorical variables are reported as frequencies and proportions. Analytical statistics, including *t*-tests and Z-tests, were used to compare variables between groups, with a *p*-value < 0.05 considered statistically significant.

Time-series analyses were also conducted using linear regression to assess trends in antibiotic prescribing over the study period; the slope of the regression line was used to define the linear trend. Antibiotic consumption was expressed as DDDs/1000 patient-days for the time-series analysis, as per the following formula provided by the WHO [31]:

$$DDDs/1000\;patient\;days=\;\frac{{DDD}_{total}\;\times \;1000/365}{N}$$

*DDD_total_* is the total amount of antibiotics prescribed in a given calendar year for the patient group, and *N* is the total number of patients in the indication category during that year.

To the best of our knowledge, no comparable long-term study has been reported from India, and such detailed prescribing data are scarce nationwide. Published studies in this field are largely short-term, cross-sectional investigations with limited ability to evaluate trends over time [34,35]. Therefore, longitudinal data gaps, particularly in low- and middle-income countries, enhance the data’s scientific value. As such, these results offer baseline evidence on prescribing practices, which are otherwise grossly missing, and can inform future studies, support comparisons over time, and contribute to the development and evaluation of antimicrobial stewardship interventions and policies.

## 5. Conclusions

In both hospitals, the number of antibiotics prescribed increased over the 10-year period. Antibiotics were also prescribed to patients in both the TH and the NTH who had no recorded bacterial infection or reason for antibiotic prophylaxis. Additionally, broad-spectrum antibiotics were prescribed to the majority of inpatients in both study hospitals. Such prescribing practices can fuel the development of ABR. The prescribing patterns highlighted a focus on quinolones, and particularly fluoroquinolones, which are from the *Watch* group of the AWaRe classification. The *Watch* group has an increased risk of becoming resistant to antibiotics, thereby increasing the risk of ABR. Thus, the use of *Watch* antibiotics must be reduced and replaced by antibiotics from the *Access* group.

The high proportion of oral systemic antibiotic use relative to topical formulations, the high antibiotic prescribing across diagnosis categories, including non-infectious indications regardless of infection status, the limited use of microbiological culture guidance, and the increasing trend in Watch antibiotic prescribing collectively suggest a stewardship gap from an antibiotic resistance perspective. A high proportion of prescriptions were recorded for non-infectious and non-surgical diagnosis categories, raising questions about the balance between prophylactic intent and therapeutic need that warrant prospective investigation. These prescribing patterns risk increasing ABR locally and contributing to the global ABR burden.

These findings underscore the urgent need to develop contextualised antibiotic prescribing guidelines for ophthalmology departments in private-sector LMIC settings, implement antibiotic stewardship programmes with active prescriber engagement, and strengthen microbiology laboratory capacity. Guidelines should be grounded in local resistance data, aligned with WHO AWaRe targets, and developed in collaboration with ophthalmology specialists to ensure clinical relevance and uptake.

## Data Availability

Data is stored under the supervision of the institutional ethics committee in accordance with institutional policy. This is to ensure the electronic security of the data and to protect the patient’s confidentiality. All interested researchers can request access to anonymous data from the Chairperson of the Ethics Committee, R.D. Gardi Medical College, Agar Road, Ujjain, Madhya Pradesh, India 456006 (email: iecrdgmc@yahoo.in, uctharc@bsnl.in), providing all details of the article. The ethical approval numbers 41/2007 and 114/2010 must be quoted with the request.

## Supplementary Materials

The following supporting information can be downloaded at: https://www.mdpi.com/article/10.3390/antibiotics15080746/s1, Figure S1: Flow chart documenting the selection of patients for the analyses; Table S1: Distribution of all antibiotics prescribed in the ophthalmology inpatients of a teaching hospital (A) and a non-teaching hospital (B) in Central India.
