# Supplementary material for: Antibiotic Prescribing Among Inpatients with Ophthalmic Diagnoses: A Decade of Evidence from Two Private-Sector Indian Hospitals
Source: Antibiotics (Basel). 2026 Jul 31;15(8):746. doi: 10.3390/antibiotics15080746 (PMC13509725; doi:10.3390/antibiotics15080746)
Supplement: Supplementary file 1 [file antibiotics-15-00746-s001.zip › antibiotics-4404524-supplementary.pdf]

Figure S1. Flow chart documenting the selection of patients for the analyses

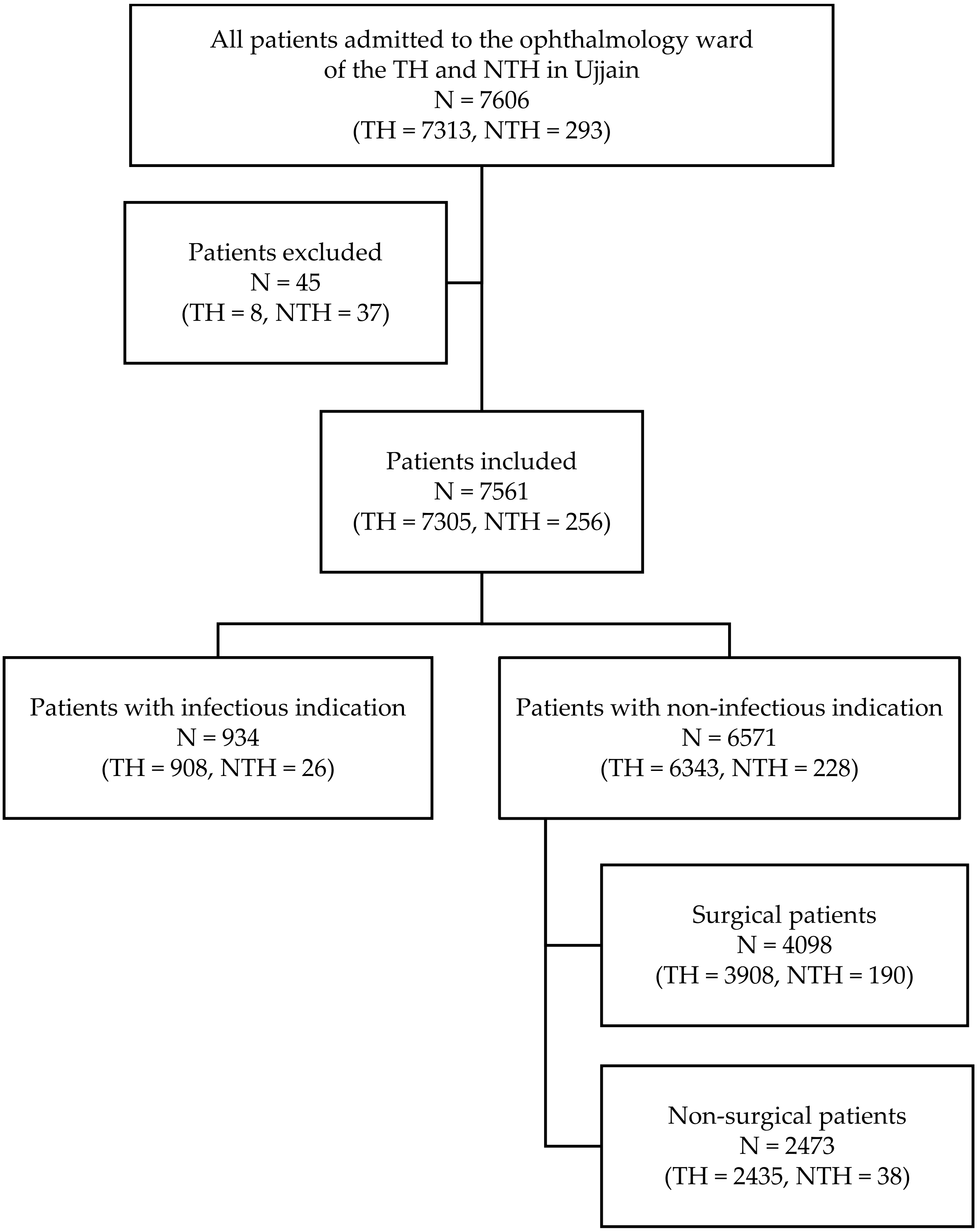

Two patients from the NTH and 54 patients from the TH had incomplete records; therefore, these patients were excluded from classification into the infectious and non-infectious categories.

Tables S1: Distribution of all antibiotics prescribed in the Ophthalmology Inpatients of a teaching hospital (A) and a Non-teaching hospital (B) in Central India.

Table S1

A- Teaching Hospital

ATC code (full)	Antibiotic name	AWaRe	Number of antibiotics prescribed (days)	Percentage of total prescriptions
J01MA02	Ciprofloxacin	Watch	42636	87.45
J01MA14	Moxifloxacin	Watch	4224	8.66
J01GB03	Gentamicin	Access	423	0.87
J01DD01	Cefotaxime	Watch	356	0.73
J01CR02	Amoxicillin + Clavulanic acid	Watch	223	0.46
J01DD08	Cefixime	Watch	151	0.31
J01CA04	Amoxicillin	Access	141	0.29
J01DB05	Cefadroxil	Access	117	0.24
J01AA02	Doxycycline	Access	102	0.21
J01DD04	Ceftriaxone	Watch	102	0.21
J01DD51	Moxifloxacin + Methylcellulose	Watch	47	0.10
J01XD01	Metronidazole	Access	51	0.08
J01DB04	Cefazolin	Access	32	0.07
J01EE01	Sulfamethoxazole + Trimethoprim	Access	30	0.06
J01CR05	Penicillin + Tazobactam	Watch	15	0.03
J01CR50	Ampicillin + Cloxacillin	Watch	15	0.03
J01XX08	Linezolid	Reserve	13	0.03
J01DD13	Cefpodoxime	Watch	10	0.02
J01DD62	Cefoperazone + Sulbactam	Watch	8	0.02
J01MA06	Norfloxacin	Watch	7	0.01
J01DC10	Cefprozil	Watch	6	0.01
J01RA13	Norfloxacin + Tinidazole	Watch	6	0.01
J01DC02	Cefuroxime	Watch	5	0.01
J01GB06	Amikacin	Watch	5	0.01
J01CA01	Ampicillin	Access	4	0.01
J01GB07	Netilmicin	Access	4	0.01
J01MA01	Ofloxacin	Watch	4	0.01
J01GB01	Tobramycin	Watch	3	0.01
J01MA15	Gemifloxacin	Watch	3	0.01
J04AB02	Rifampicin	NC†	3	0.01
J04AC01	Isoniazid	NC†	3	0.01
J04AK01	Pyrazinamide	NC†	3	0.01
J04AK02	Ethambutol	NC†	3	0.01

B- Non-teaching Hospital

ATC code (full)	Antibiotic name	AWaRe	Number of antibiotics prescribed on the ward (days)	% of total prescriptions
J01MA02	Ciprofloxacin	Watch	178	52.82
J01MA14	Moxifloxacin	Watch	74	21.96
J01DD08	Cefixime	Watch	21	6.23
J01DD13	Cefpodoxime	Watch	21	6.23
J01GB01	Tobramycin	Watch	7	2.08
J01CR02	Amoxicillin + Clavulanic acid	Watch	6	1.78
J01DD04	Ceftriaxone	Watch	5	1.48
J01MA16	Gatifloxacin	Watch	5	1.48
J01DB01	Cefalexin	Access	4	1.19
J01DD63	Ceftriaxone + Beta-lactamase inhibitor	Watch	3	0.89
J01DC02	Cefuroxime	Watch	2	0.59
J01XD01	Metronidazole	Access	2	0.59
J01CA04	Amoxicillin	Access	1	0.30
J01DB04	Cefazolin	Access	1	0.30
J01GB03	Gentamicin	Access	1	0.30
J01GB06	Amikacin	Watch	1	0.30
J01MA01	Ofloxacin	Watch	1	0.30
J01MA12	Levofloxacin	Watch	1	0.30
J01RA13	Norfloxacin + Tinidazole	Watch	1	0.30
J01RA91	Cefixime + Ofloxacin	Watch	1	0.30
J04AM06	Rifampicin, Pyrazinamide, Ethambutol + Isoniazid	NC†	1	0.30

† TB medications (rifampicin, isoniazid, pyrazinamide, ethambutol) are not included in the WHO AWaRe Classification and are therefore not classified. Abbreviations: ATC, Anatomical Therapeutic Chemical; AWaRe, Access, Watch, Reserve (WHO 2022 Antibiotic Classification); DDD, Defined Daily Dose; NC, not classified; NTH, Non-teaching Hospital; TH, Teaching Hospital.
